# Analyzing the distribution of virulence factors of *Mycobacterium tuberculosis* and the impact of virulence gene mutations on treatment outcomes in different lineages using whole-genome sequencing in Urumqi

**DOI:** 10.1080/21505594.2025.2552875

**Published:** 2025-09-16

**Authors:** Jiandong Yang, Yaoqin Lu, Yanggui Chen, Yida Wang, Chao Wang, Kai Wang

**Affiliations:** a School of Public Health, Xinjiang Medical University, Urumqi, People’s Republic of China; b Department for Tuberculosis Control and Prevention, Urumqi Center for Disease Control and Prevention, Urumqi, People’s Republic of China; c Laboratory of Pathogenesis, Prevention and Treatment of High Incidence Diseases in Central Asia, Department of Medical Engineering and Technology, Xinjiang Medical University, Urumqi, China

**Keywords:** *Mycobacterium tuberculosis*, virulence factors, mutation, whole-genome sequencing

## Abstract

This study centers on Urumqi, utilizing whole-genome sequencing and comparative genomics, we explored virulence factor mutations in different *Mycobacterium tuberculosis* lineages and their impact on tuberculosis patient prognosis. We utilized routine national drug resistance surveillance data from Urumqi, gathering demographic, epidemiological, and clinical data of patients with tuberculosis between 1 January 2017 to 31 December 2021. Whole-genome sequencing was employed, followed by bioinformatics analysis using various methods and statistical models. A total of 457 patients with tuberculosis were analyzed. Through whole-genome sequencing and bioinformatics analysis, we categorized these strains into three lineages: Lineage2 (347), Lineage3 (37), and Lineage4 (73), identifying 71 virulence factor mutations. The mutation rates of virulence factors in *M. tuberculosis* exhibited polarization. Significant differences in virulence factor mutation rates were observed among different *M. tuberculosis* lineages (all *p* values  < 0.05). Additionally, mutations in espE, fadE29, and mbtI genes among Lineage2 patients were considered as risk factors influencing treatment outcomes (all *p* values < 0.05), with odds ratios of 13.6200 (1.7285–107.3201), 7.1262 (1.3294–38.1997), and 14.8340 (1.1577–190.0784), respectively. Varied virulence factor mutations and virulence factor-related gene mutations exist across different *M. tuberculosis* lineages. Mutations in the espE, fadE29, and mbtI genes are risk factors that significantly affect the treatment outcome of Lineage2 patients. This finding serves as a reference for investigating the future evolutionary direction, transmissibility, drug resistance, and pathogenicity of *M. tuberculosis* virulence factors in regions with diverse lineages and frequent population movements.

## Background

Tuberculosis has been a persistent threat to human health across history [1–3]. Our understanding of tuberculosis has evolved from identifying patient symptoms to exploring the microscopic morphology and molecular characteristics of *Mycobacterium tuberculosis* [4]. Recent advancements in molecular biology have facilitated deeper investigations into *M. tuberculosis* at the molecular level, notably in studying its virulence factors. Unlike most pathogens, *M. tuberculosis* does not rely on pili, flagella, and toxins for host invasion or transmission [5]. Instead, it thrives by triggering strong inflammation and lung tissue destruction, relying on its adeptness in manipulating host macrophages to hide and evade the immune response [6]. This process involves the participation of various virulence factors, including ESAT-6, sigE, and ESX-1 to ESX-5 [7,8].

Studies on the virulence factors of *M. tuberculosis* and their role in human host invasion and disease fall into three main categories: secretion systems, bacterial components, and related enzymes. The secretion system (ESX-1 to ESX-5) of *M. tuberculosis* transports various virulence factors across the host cell envelope, influencing the host immune response, disrupting cell integrity, and eventually leading to cell death [9,10]. While the importance of the secretion system in *M. tuberculosis* pathogenesis is well-established, its structural specifics warrant further exploration. Another critical class of virulence factors for *M. tuberculosis* is lipids. *M. tuberculosis* can synthesize various atypical lipids that are exposed on the cell surface, which assist in infecting macrophages and evading host cell immune response [11]. The main lipid virulence factor of *M. tuberculosis* is Phthiocerol dimycocerosate (DIM/PDIM), which promotes the entry of *M. tuberculosis* into macrophages through phagocytosis. However, its molecular mechanism of action remains unknown [12]. Additionally, enzymes, like IpdAB, part of the CoT superfamily, are closely related to *M. tuberculosis* pathogenicity due to their involvement in steroid ring hydrolysis, a process vital for the pathogen’s reliance on host-derived cholesterol. However, further research is needed to determine the specific mechanism [13]. Additionally, other factors such as *M. tuberculosis* cell wall components, polysaccharides, and special proteins facilitate *M. tuberculosis* invasion and disease but are currently under-researched [14–17].

Advancements in whole-genome sequencing technology and comparative genomics have enabled researchers to study *M. tuberculosis* virulence factors. Variances in invasiveness and disease-causing capabilities of the same virulence factor across different *M. tuberculosis* lineages have been observed [18–20]. Among these lineages, Lineages 2 and 4 are the most prevalent globally. The modern Beijing genotype of Lineage2 is characterized by high virulence and drug resistance, while Lineage 4 exhibits extensive transmissibility. Moreover, other lineages display varying degrees of geographic isolation [21–26]. The different characteristics of these lineages can be attributed to the evolution of *M. tuberculosis* virulence factors in response to the different human hosts in various regions. This evolution has facilitated the adaptation of *Mycobacterium tuberculosis* to human hosts in different regions [27–29]. Some researchers argue that regions where multiple lineages coexist provide favorable conditions for the flow and genetic drift of dominant genes among different *M. tuberculosis* lineages, offering opportunities for further development of virulence factors through adaptive evolution [2,30]. While research on gene flow among different lineages of *M. tuberculosis* is currently limited, it is evident that this gene flow plays a crucial role in the evolution of its virulence factors. Additionally, it offers insights for the development of novel anti-tuberculosis drugs.

Historically, *M. tuberculosis*, originating from Africa, has spread globally through population movements and trade [31–33]. China, one of the countries with the highest tuberculosis burden, has seen the disease arrive via three primary routes: Russian Far East (entering northeastern China and then spreading to China’s inland and coastal areas); Maritime Silk Road (entering inland areas through the southeastern coast); and the land Silk Road through Xinjiang (reaching both inland and coastal areas) [34–36]. Similarly, *M. tuberculosis* from China can also spread to other parts of the world through these paths, resulting in a two-way transmission process [37]. Xinjiang, a province in northwestern China, is among the provinces with a high burden of tuberculosis [38,39]. Xinjiang has long served as a vital transportation hub connecting China to Central Asia and Europe, playing a crucial role in trade and serving as a trade transfer station on the Silk Road. This unique geographical location, coupled with the constant movement of people and thriving trade, has facilitated the spread of *M. tuberculosis* and the gene flow of various lineages of the bacterium [40,41]. Notably, geographical areas with multiple lineages coexisting are more likely to experience gene flow and drift, which are key forces behind the variation and evolution of virulence factors. Therefore, understanding virulence factors in regions with diverse lineages and high population mobility is crucial to forecasting the evolution, transmissibility, drug resistance, and pathogenicity of *M. tuberculosis*. Additionally, such insights are pivotal for global tuberculosis prevention and control efforts, aligning with the World Health Organization’s (WHO) *2035 goal of eliminating tuberculosis*. This study concentrates on Urumqi, Xinjiang, a hub of frequent population movements and trade along the Silk Road’s core economic belt. Whole-genome sequencing technology and comparative genomics methods were employed to investigate the differences in virulence factor mutations across the various lineages of *M. tuberculosis* and their impact on the prognosis of patients with tuberculosis.

## Information and methods

As part of our broader research on regional tuberculosis, this study is generally consistent with our previous work in terms of data collection, sample sources, experimental methodologies, and bioinformatics analysis approaches, among other aspects [42]. Details are as follows:

### Data source and database establishment

This retrospective study utilized routine national drug resistance surveillance data from Urumqi. Over 5 years (1 January 2017 to 31 December 2021), 457 MTB strains from sputum samples of suspected patients with tuberculosis who visited eight tuberculosis sentinel hospitals in Urumqi City were obtained and subjected to whole-genome sequencing (WGS). Subsequently, we comprehensively analyzed the distribution of virulence factors in *M. tuberculosis* and evaluated the impact of mutations in virulence genes on treatment outcomes across different lineages. Informed consent was obtained from all participants (minors were excluded).

In order to ensure that the sample size can meet the minimum sample size required for statistical analysis, we conducted sample size estimation, as follows: Since this study as a whole is a current situation survey, the sample size calculation formula of the current situation survey is used to estimate the sample size.

$$N=\frac{\mu_{\alpha }^{2}\times \pi \times \left(1-\pi \right)}{\delta^{2}}\times \left(1+K\right)$$

The significance level (*α*) is 0.05 for both sides, and the allowable error (*δ*) is 0.05. According to monitoring data from the Tuberculosis Reference Laboratory of the Urumqi Center for Disease Control and Prevention, the positive rate of *Mycobacterium tuberculosis* culture in Urumqi is approximately 24.86%. Therefore, π is equal to 24.86%. Since patients may not respond during sample collection, this study defines this proportion as K, with a value of 10%. Plugging the above values into the above formula, the final calculated sample size N is approximately 316. Therefore, the sample size (416) of this study was larger than the estimated sample size (316). The sample number was sufficient to meet the minimum sample size required for statistical analysis.

### Patient information

Basic patient data were sourced from the National Drug Resistance Surveillance Database (NDRS). This database collects information through interviews conducted by doctors at the eight TB sentinel hospitals in Urumqi during patient visits.

### Strain isolation and culture and strain identification

MTB strains were isolated and cultured using a solid culture method for mycobacteria. Sputum samples underwent pretreatment with 4% NaOH before inoculation into acidic Lowenstein-Jensen medium (L-J medium). The cultures were then incubated at a constant temperature of 37°C. The strains were identified using P-Nitrobenzoic acid and Thiophen-2-carboxylic acid hydrazide identification medium. Detailed procedures and interpretation are outlined in Appendices S1 and S2.

### Extraction of Mycobacterium DNA and WGS

Mycobacterium DNA extraction utilized the manual membrane adsorption column method and the PureLink™ microbiome DNA purification kit from Thermo Fisher Scientific. The extracted DNA underwent quality assessment via agarose gel electrophoresis and quantification using Qubit. DNA samples with grade D results were excluded. Details are provided in Appendices S3 and S4.

DNA samples were fragmented into approximately 350 bp fragments using a Covaris ultrasonic breaker. The resulting DNA fragments were processed using the NEBNext®Ultra™ DNA Library Prep Kit for Illumina (NEB, USA), involving terminal repair, A tail addition, sequencing adapter addition, purification, and PCR amplification. The library type established was a 350-bp small fragment library. After library construction, preliminary quantification was performed using Qubit 2.0. The library was diluted to a concentration of 2ng/ul, and the inserted fragments were detected using the Agilent 2100 system. Once the insert size met the expected range, the library’s effective concentration was accurately quantified using Quantitative Real-Time Polymerase Chain Reaction (qPCR) to ensure its quality. Finally, the Illumina PE150 sequencing platform was used for on-machine sequencing.

### Mapping and variant calling

Quality control of obtained FASTQ files was executed using FastQC v0.11.9 (https://www.bioinformatics.babraham.ac.uk/projects/fastqc/), followed by low-quality fragment processing using Trimmomatic v0.39 (http://www.usadellab.org/cms/index.php?page=trimmomatic). H37Rv (NC_000962.3) was used as the reference sequence, and Snippy v4.6.0 software (https://github.com/tseemann/snippy) was used for variant detection, core genome alignment, and SNP detection. Furthermore, snippy-core (a subtool of the snippy software) was utilized for SNP merging. GATK4 v4.0.4.0 software (https://gatk.broadinstitute.org) and Vcftools software (http://vcftools.sourceforge.net/) were used for base quality filtering and group labeling quality filtering. The filtered data was used in the subsequent analyses.

### Phylogenetic reconstruction

A total of 95% of SNP positions were aligned, excluding SNPs in repetitive regions such as PE/PPE-PGRS family genes, insertions, mobile elements, or phage sequences. Maximum likelihood evolutionary trees were constructed using IQ-TREE v2.2.2.7 software (http://www.iqtree.org/), employing ModelFinder Plus (MFP), a sub-command of IQ-TREE, with Bayesian methods, for best-fit model selection. The bootstrap method was applied 10,000 times. The ggtree package in RStudio Desktop v2022.07.1–554 software (https://www.rstudio.com/products/rstudio/download/) facilitated tree visualization.

### Lineage and virulence factors

The TB-Profiler v4.4.2 software (https://github.com/jodyphelan/TBProfiler) was used for lineage identification based on whole-gene sequencing, while Abricate software (https://anaconda.org/bioconda/abricate) identified virulence factors.

### Statistical analysis

We use composition ratios or rates to describe the data. According to the data distribution type, we choose the method suitable for the data type among the three methods *χ2* test, adjust *χ2* test, Fisher’s exact test to conduct distribution difference analysis and single-factor analysis of influencing factors. Multi-factor logistic regression model and generalized multi-factor dimensionality reduction model (GMDR) were used to conduct multi-factor analysis of influencing factors and gene–gene interaction analysis. The logistic regression model uses the forced entry method and assigns values to categorical variables. Generalized multi-factor dimensionality reduction model uses cross-validation consistency for model evaluation. In addition, a logistic regression model was used to construct a drug resistance gene propensity score, and a t test was used to analyze the distribution of drug resistance gene propensity scores in the cured and uncured groups. We use RStudio Desktop v2022.07.1–554 software (https://www.rstudio.com/products/rstudio/download/) and GMDR software (http://ibi.zju.edu.cn/software/GMDR/download.html) to implement the above analysis process. In addition, we use RNArtist (https://github.com/fjossinet/RNArtist) and SWISS-MODEL (https://swissmodel.expasy.org/) to predict and construct nucleotide secondary structure and protein secondary structure, respectively, use PROVEAN PROTEIN (http://provean.jcvi.org/seq_submit.php) to predict protein function, and use PyMOL (https://pymol.org/) for visualization. A significance level of α = 0.05 was used, and differences were considered statistically significant when *p* < 0.05.

## Results

### Basic information

A total of 457 patients with tuberculosis participated in this study, with a higher representation of men (59.96%) than women (40.04%). The majority of patients (39.61%) were aged 65 and above. Patient occupations can be categorized into six groups: farmers or herders, service industry personnel, retirees, students, medical staff, and others. Among these, the service industry had the highest representation (38.51%), followed by retirees (28.01%). In terms of residence, urban areas (86.00%) housed the majority of patients, predominately non-migrants (77.90%). Analysis of the classification of patients and treatment outcomes revealed that 86.87% of the patients were treatment-naïve, while 13.79% experienced treatment failure, including cases transferred to multidrug-resistant treatment, lost follow-up, and death due to other diseases. In addition, all 457 patients involved in this study had a history of BCG vaccination and 2 patients had complications (malnutrition), and all patients did not use immunosuppressants during treatment. Further details can be found in Table 1 and Supplementary S1.

**Table 1.** Characteristics of patients with TB.

Variables		Count (N = 457)	Percentage (%)
Sex			
	Male	274	59.96
	Female	183	40.04
Age			
	<20	19	4.16
	20–35	85	18.60
	35–65	172	37.64
	≥65	181	39.61
Occupation			
	Farmers and herdsmen	63	13.79
	Service workers	176	38.51
	Retiree	128	28.01
	Students	16	3.50
	Medical workers	4	0.88
	Other	70	15.32
Residence			
	Rural	64	14.00
	Urban	393	86.00
Floating population			
	Yes	101	22.10
	No	356	77.90
Classification			
	Initial treatment	397	86.87
	Re-treatment	60	13.13
Treatment result			
	Cured	394	86.21
	Not cured	63	13.79
Complication			
	Diabetes mellitus	0	0.00
	Malignant tumor	0	0.00
	Malnutrition	2	0.44
	Other complication	0	0.00
	Not complication	456	99.56
BCG vaccination history			
	Yes	457	100.00
	No	0	0.00
Whether immunosuppressants			
	Yes	0	0.00
	No	457	100.00

Describe the basic situation of the patient through numbers and composition comparisons.

### Analyzing mutations in virulence factors of M. tuberculosis among various lineages

**Figure 1.**
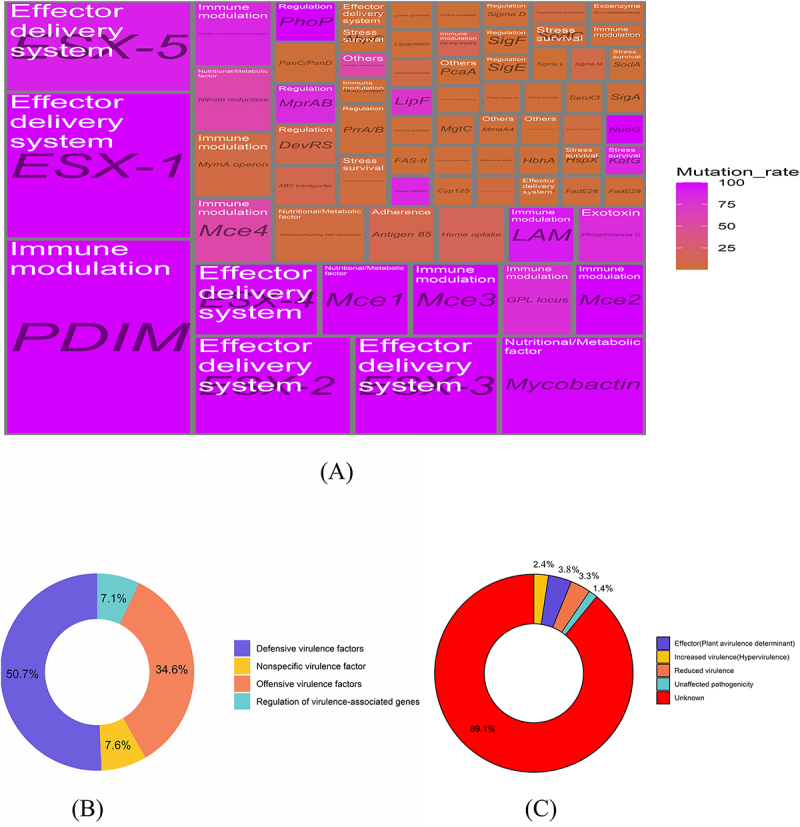
Distribution of virulence factors and virulence factor-related genes. (A) A rectangular tree diagram depicting virulence factors, virulence factor-related genes, and virulence factor mutation rates; (B) A diagram classifying virulence factor-related genes based on the virulence factor database; (C) A composition map of genes related to virulence factors classified according to the pathogen host interactions database.

Comparative analysis with the Virulence Factor Database (VFDB) database revealed 71 virulence factors, covering 85.54% of virulence factors compared to the standard strain of H37Rv’s 83 virulence factors. Among these, 23 virulence factors exhibited mutation rates above 56.24%, while 48 had mutation rates below 16.19%. Notably, nine virulence factors (ESX-1, ESX-2, ESX-3, Mce1, Mce2, Mce3, NuoG, PDIM, and PhoP) exhibited a 100.00% mutation rate. Furthermore, analysis of 27 virulence factors across *M. tuberculosis* lineages exhibited statistically significant differences in mutation rates (*p* values  < 0.05). The mutation rates of virulence factors LipF and Mce4 in Lineage2 were observed to be at least twice as high as those in Lineage3 and Lineage4. Additionally, the mutation rates of virulence factors ABC transporter, Antigen 85, DevRS, Lipid phosphatase, and PE/PE-PGRS were three times higher in Lineage3 compared to Lineage2 and Lineage4. Similarly, the mutation rate of virulence factors Nitrate/nitrite transporter, Tryptophan synthesis, and Tyrosine phosphatase exhibited three times the rate in Lineage4 than in Lineage2 and Lineage3. The mutation rates of 71 virulence factors showed differences among different lineages. Furthermore, our analysis revealed that all Lineage2 strains belonged to the Beijing family, with a higher mutation rate (60.56%) observed in L2.2.1 compared to L2.2.2. Further details are available in Figure 1 and Supplementary S2-S4.

### Analyzing mutations in related genes of virulence factors of M. tuberculosis among various lineages

**Figure 2.**
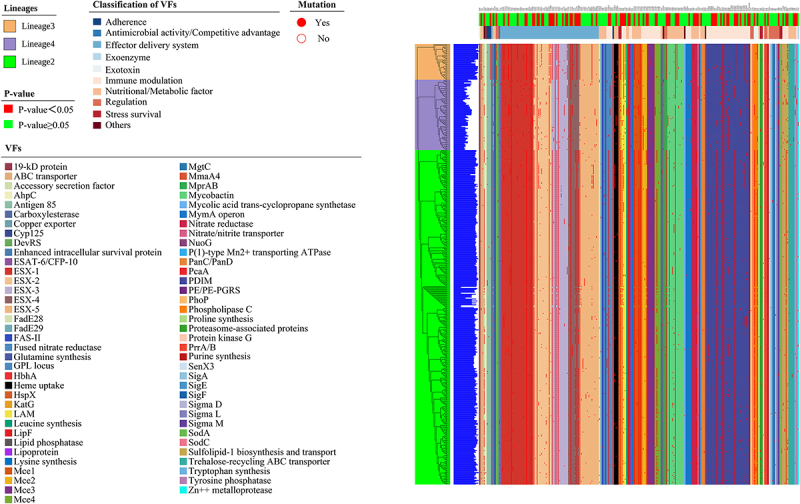
Phylogeny of 457 *Mycobacterium tuberculosis* strains. The color on the evolutionary tree represents the lineage to which it belongs. The blue graphics in the second vertical column indicates the mutated number of virulence genes, while the third vertical column shows which virulence factor the 211 virulence genes belong to. The red circle indicates the occurrence of mutations in virulence genes, with hollow circles indicating no mutations in virulence genes. The first horizontal row indicates whether there is a difference in the distribution of virulence factors between the treatment success group and the failure group, while the second horizontal row indicates the classification of virulence factors.

According to the classification standard of VFDB, a total of 211 virulence genes were divided into four categories: defensive virulence factors, offensive virulence factors, regulation of virulence-associated genes, and nonspecific virulence factors. Defensive virulence factors accounted for the highest proportion (50.71%), followed by offensive virulence factors (34.60%), while regulation of virulence-associated genes had the smallest proportion (7.11%). Additionally, based on the Pathogen Host Interactions-base (PHI-base) database, the proportions of increased virulence (hypervirulence), effector (plant avirulence determinant), reduced virulence, unaffected pathogenicity, and unknown were 2.37%, 3.791%, 3.32%, 1.42%, and 89.10%, respectively. Upon analyzing the mutation rate of genes, we observed that the nuoG gene in Lineage2 and Lineage3; eccE3 gene and PE35 gene in Lineage3 and Lineage4; and the eccA2 gene, eccA3 gene and mycP4 gene in Lineage3 exhibited a 100% mutation rate. Furthermore, we examined the variation in mutation rates of the 211 genes across different lineages. Statistically significant differences (all *p* values < 0.05) were observed in the mutation rates of 86 genes among the three lineages (Lineage2, Lineage3, and Lineage4). In Lineage2, the mutation rates of 33 genes related to virulence, including PE5, PPE4, and PPE41, were equal to or higher than those observed in Lineage3 and Lineage4. Similarly, in Lineage3, 33 virulence factor-related genes with mutation rates equal to or higher than those in Lineage2 and Lineage4 were observed. However, in Lineage4, only 20 genes related to virulence factors demonstrated mutation rates that were equal to or surpassed those observed in Lineage2 and Lineage3. Elaborated data are presented in Figures 1–2 and Supplementary S2, S5–S6.

### Impact of mutations in virulence factor-related genes on the outcome of patients with tuberculosis

**Figure 3.**
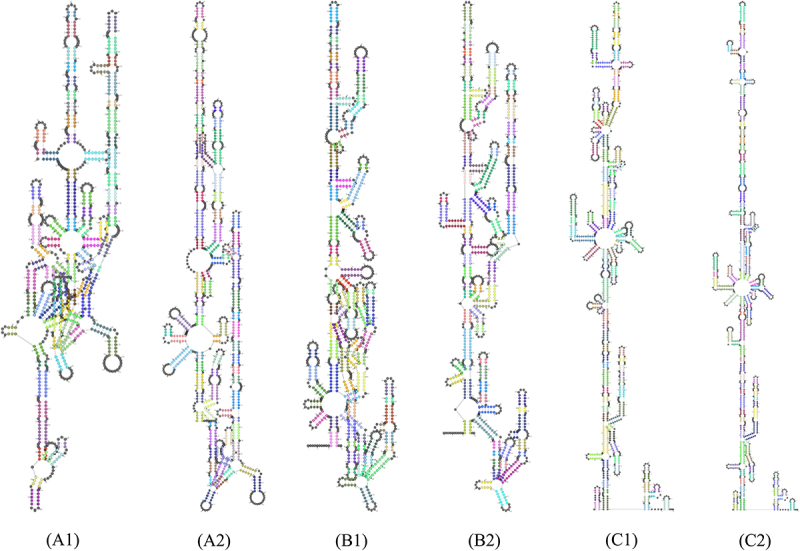
Predicted nucleotide secondary structures of espE gene, fadE29 gene, and mbtI gene. (A1), (B1), and (C1) are the secondary structures of nucleotides of wild-type espE gene, fadE29 gene, and mbtI gene, respectively. (A2), (B2), and (C2) are the secondary structures of nucleotides of mutant-type espE gene, fadE29 gene, and mbtI gene respectively. Different colors in the figure represent different nucleotide secondary structures.

**Figure 4.**
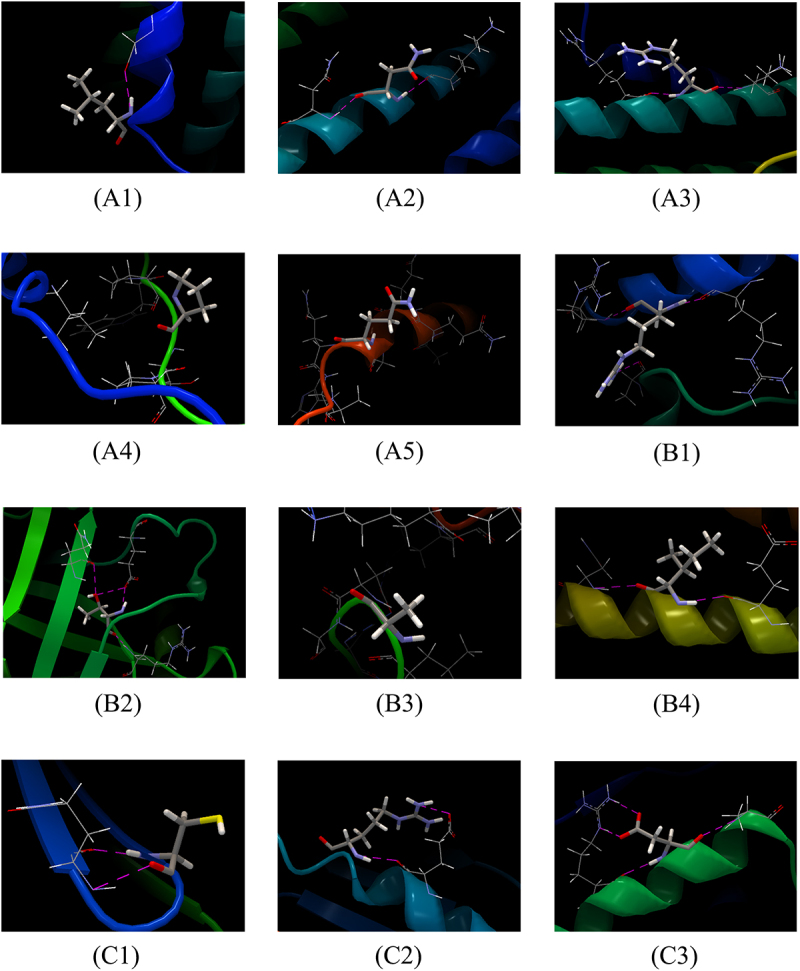
Interactions between the amino acids at wild-type sites corresponding to the mutation sites of espE gene, fadE29 gene, and mbtI gene and surrounding amino acids. (A1)–(A5), (B1)–(B4), and (C1)–(C3) are the interactions between the amino acids at the wild-type site corresponding to each mutation site of espE gene, fadE29 gene, and mbtI gene and surrounding amino acids, respectively. (A1)-(A5) are Leu21, Asn87, Arg104, Pro223 and Gln362 of the espE gene respectively. (B1)-(B4) are Arg46, Thr139, Ala202, and Ile288 of the fadE29 gene, respectively. (C1)–(C3) are Cys50, Arg98, and Asp162 of the mbtI gene, respectively. In the figure, the stick represents the target amino acid, the wireframe represents the amino acids surrounding the target amino acid, and magenta dotted lines denote hydrogen bonds with the surrounding amino acid residues.

**Figure 5.**
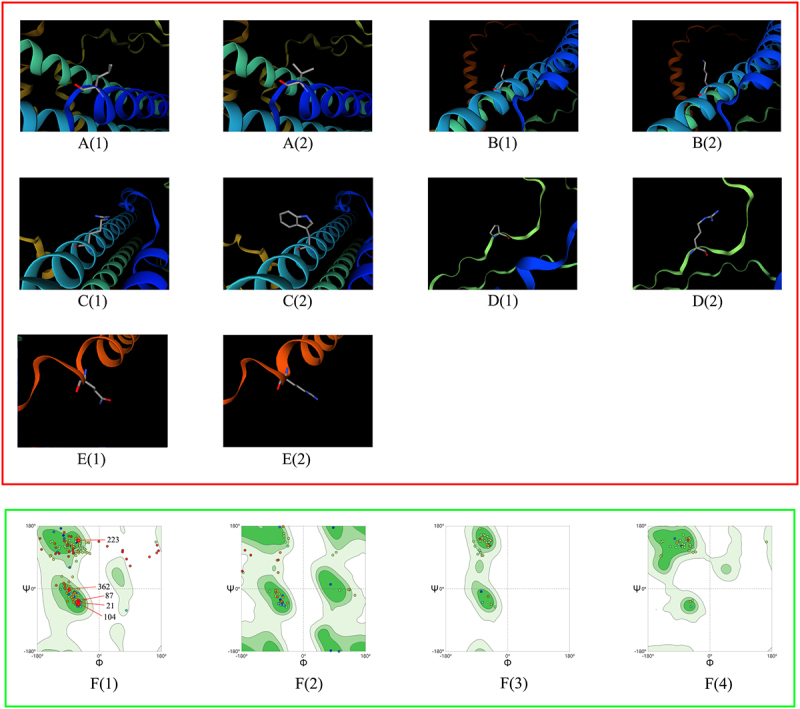
Predicted three-dimensional protein structure diagram of each mutation site of espE gene. The red box in the figure is the protein structure diagram, and the red box is the ramachandran plots. A(1), B(1), C(1), D(1), and E(1) are the wild-type protein structure diagrams at positions 21, 87, 104, 223, and 362, respectively, and A(2), B(2), C(2), D(2), and E(2) are the mutant-type protein structure diagrams of the above-mentioned positions respectively. F(1) to F(4) are the general Rasperchandr diagram, glycine Rasperchandr diagram, proline Rasperchandr diagram, and pre-proline Rasperchandr diagram, respectively. It can be seen from the Rasperchandr diagram that the predicted structures are relatively reasonable and are all located in dark areas.

**Figure 6.**
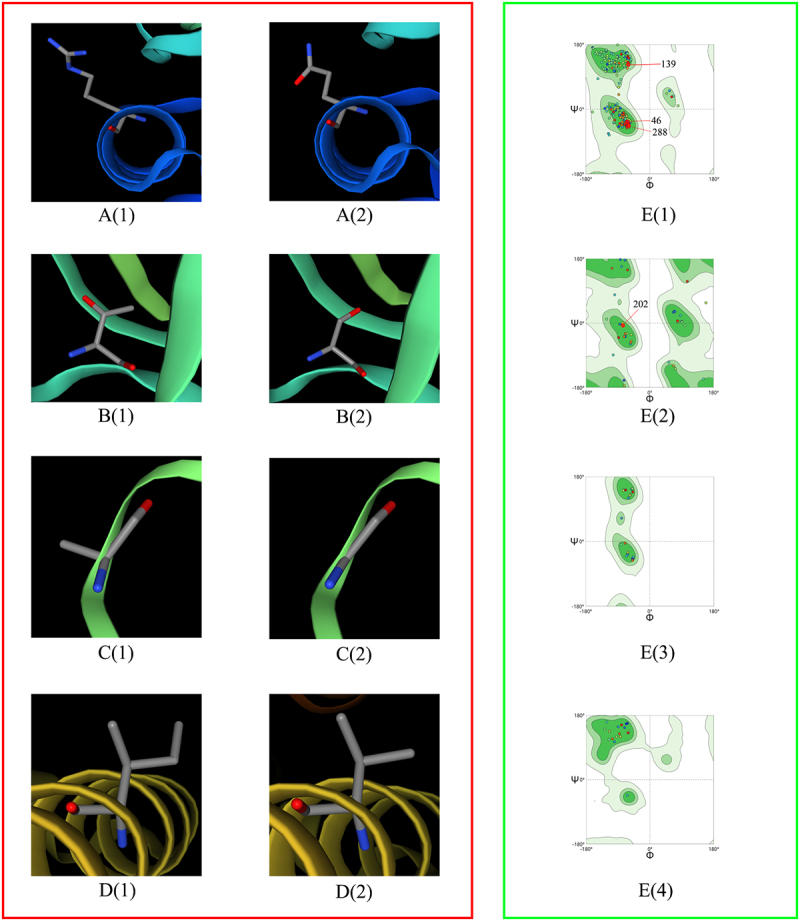
Predicted three-dimensional protein structure diagram of each mutation site of fadE29 gene. The red box in the figure is the protein structure diagram, and the red box is the Ramachandran plots. A(1), B(1), C(1), and D(1) are the wild-type protein structure diagrams at positions 46, 139, 202, and 288, respectively, and A(2), B(2), C(2), and D(2) are the mutant-type protein structure diagrams of the above-mentioned positions, respectively. E(1) to E(4) are the general Rasperchandr diagram, glycine Rasperchandr diagram, proline Rasperchandr diagram, and pre-proline Rasperchandr diagram, respectively. It can be seen from the Rasperchandr diagram that the predicted structures are relatively reasonable and are all located in dark areas.

**Figure 7.**
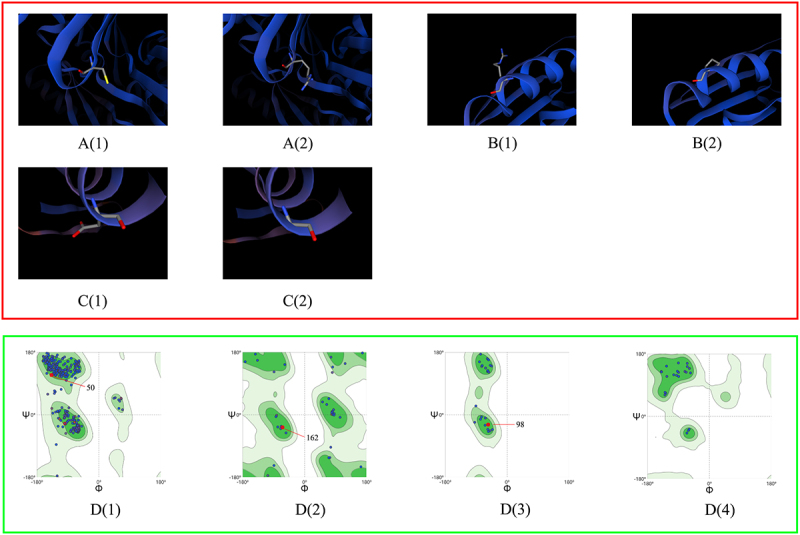
Predicted three-dimensional protein structure diagram of each mutation site of mbtI gene. The red box in the figure is the protein structure diagram, and the red box is the ramachandran plots. A(1), B(1), and C(1) are the wild-type protein structure diagrams at positions 50, 98, and 162, respectively, and A(2), B(2), and C(2) are the mutant-type protein structure diagrams of the above-mentioned positions, respectively. D(1) to D(4) are the general Rasperchandr diagram, glycine Rasperchandr diagram, proline Rasperchandr diagram and pre-proline Rasperchandr diagram respectively. It can be seen from the Rasperchandr diagram that the predicted structures are relatively reasonable and are all located in dark areas.

To investigate the influence of virulence factor-related gene mutations (Mutation types include SNP, MNP, insertions, deletions, and complex variation) in different lineages on patient treatment outcomes, we analyzed 211 virulence factor-related genes and their association with patient treatment outcomes. Considering that the proportion of patients with multidrug-resistant tuberculosis is higher in the uncured group, drug-resistant gene mutations may affect the relationship between virulence-related gene mutations and patient treatment outcomes, leading to bias. To truly reflect the relationship between virulence-related gene mutations and treatment outcomes of tuberculosis patients, we used the propensity score method to analyze the distribution of resistance gene mutations of anti-tuberculosis drugs that need to be monitored in China`s national tuberculosis drug resistance surveillance project between the cured group and the not cured group. These drugs include Isoniazid (INH), Rifampicin (RFP), Streptomycin (SM), Amikacin (AM), Kanamycin (KM), Capreomycin (CM), Ethambutol (EMB), Fluoroquinolones (include Ofloxacin (OFX), Levofloxacin (LEV), and Moxifloxacin (MXF)), and Para-aminosalicylic acid (PAS). The resistance genes to the above anti-tuberculosis drugs detected in this study include the following genes: ahpC, fabG1, inhA, katG, rpoB, rpoC, gid, rpsL, rrs, embA, embB, gyrA, gyrB, eis, folC, and thyA. The results showed that there were statistical differences in the distribution of drug resistance gene propensity scores of Lineage2 *Mycobacterium tuberculosis* between the cured group and the not cured group (*t* = −2.6174, *p* = 0.0115). Therefore, drug resistance genes are likely to interfere with the results when analyzing the relationship between Lineage 2 *Mycobacterium tuberculosis* virulence-related gene mutations and treatment outcomes in patients infected with Lineage 2 *Mycobacterium tuberculosis*. However, there is no statistical difference in the distribution of drug resistance gene propensity scores of Lineage 3 (*t* = −0.822, *p* = 0.4166) and Lineage 4 (*t* = −1.9374, *p* = 0.0749) *Mycobacterium tuberculosis* between the cured group and the not cured group. This means that resistance genes will not interfere with the analysis of Lineage 3 and Lineage 4. Univariate analysis revealed an association between genes such as PPE26, espE, fadE29, lipF, mas, mbtB, mprB, mmpL10, mbtI, aftB, ppsA, eccC4, and treatment outcomes in patients infected with the Lineage2 of *M. tuberculosis*. Among them, eccC4 and ppsA are deletion variations and MNP variations, respectively, and the remaining variations are SNP variations. It is worth noting that we did not observe any difference in the distribution of insertions variation and complex variation between the cured group and the not cured group. Additionally, univariate analysis results showed that genes eccE2, purC, and PPE68 were found to influence treatment outcomes in patients infected with the Lineage3 of *M. tuberculosis*. Univariate analysis of Lineage4 *Mycobacterium tuberculosis* showed that we did not observe an impact of mutations in any virulence-related genes on outcomes in patients infected with lineage 4 *Mycobacterium tuberculosis*. Considering that when analyzing the relationship between *Mycobacterium tuberculosis* lineage 2 virulence-related gene mutations and treatment outcomes in patients with *Mycobacterium tuberculosis* lineage 2, drug resistance genes may interfere with the results. Therefore, when performing a multivariate logistic regression analysis of lineage 2 *Mycobacterium tuberculosis* virulence-related genes and treatment outcomes of tuberculosis patients infected with *Mycobacterium tuberculosis*, we included the resistance gene propensity score value as a covariate into the multifactor logistic regression model to control its impact on the results. Further multivariate logistic regression analysis on the treatment outcome of patients infected with Lineage2 *M. tuberculosis* revealed that the model was statistically significant (χ2 = 51.1585, p < 0.0001). The espE gene, the fadE29 gene, and the mbtI gene were identified as risk factors for treatment outcomes in patients infected with Lineage2 *M. tuberculosis*. Compared to the non-mutated group, the relative risk of non-cured outcomes in the mutated group was 13.6200 times, 7.1262 times, and 14.8340 times higher, respectively. To explore the interactive effects of the espE gene, fadE29 gene, and mbtI gene on the treatment outcomes of patients infected with Lineage2 *M. tuberculosis*, we used a GMDR model for interaction analysis. The results showed no interaction between these genes. The prediction results of the nucleotide secondary structure of the espE gene, fadE29 gene, and mbtI gene showed that compared with the wild type, the nucleotide secondary structure of the mutant type changed to varying degrees. The three-dimensional structure of the proteins encoded by the above three genes was predicted. The results showed that the amino acids encoded by each mutation site of the above genes are located at hydrogen bond connections (Except for P223R and Q362R of espE gene, and A202G of fadE29 gene). In addition, the prediction results of the impact of mutations at each site of the above three genes on protein function show that among the 12 mutation sites of the three genes, only R46Q and T139S of the fadE29 gene are deleterious mutations, and the rest are neutral mutations. Similarly, the same analysis on patients infected with L3 *M. tuberculosis* revealed that the model was statistically significant (χ2 = 13.9472, p = 0.0030). However, the genes eccE2, purC, and PPE68 were not statistically significant in the multivariate analysis (*p* values > 0.05). Detailed findings can be found in Tables 2–8, Figures 3–7 and Supplementary S7.

**Table 2.** Difference analysis of the distribution of propensity scores of drug-resistant genes between the cured group and the not cured group.

				Test for homogeneity of variances		t-test	
Lineages	Treatment result	*Mean* ± *SD*	*M*(*P*_*25*_,*P*_*75*_)	*F*	*P*	*t*	*P*
Lineage 2	Not cured	0.1289 ± 0.0538	0.1043(0.1043,0.1043)	23.3229	<0.0001	−2.6174	0.0115*
	Cured	0.1568 ± 0.0693	0.1043(0.1043,0.2464)				
Lineage 3	Not cured	0.1061 ± 0.0455	0.1250(0.1250,0.1250)	4.0055	0.0532	−0.822	0.4166
	Cured	0.1250 ± 0.0001	0.1250(0.1250,0.1250)				
Lineage 4	Not cured	0.1553 ± 0.0956	0.1859(0.1763,0.1859)	8.1700	0.0056	−1.9374	0.0749*
	Cured	0.2833 ± 0.2340	0.1859(0.1859,0.2548)				

*The statistical method is the adjusted t-test, and the test value is the adjusted *t* value.(Failed the homogeneity of variances test).

**Table 3.** Multivariate analysis variable assignment table of the impact of Lineage 2 *Mycobacterium tuberculosis* virulence-related genes mutations on the prognosis of tuberculosis patients.

Variable	Value
Dependent variable	Cured = 0, Not cured = 1
Independent variable	
PPE26	Wild type = 0, Mutation type = 1
aftB	Wild type = 0, Mutation type = 1
eccC4	Wild type = 0, Mutation type = 1
espE	Wild type = 0, Mutation type = 1
fadE29	Wild type = 0, Mutation type = 1
mbtB	Wild type = 0, Mutation type = 1
mas	Wild type = 0, Mutation type = 1
mbtI	Wild type = 0, Mutation type = 1
mmpL10	Wild type = 0, Mutation type = 1
mprB	Wild type = 0, Mutation type = 1
PPE26	Wild type = 0, Mutation type = 1
ppsA	Wild type = 0, Mutation type = 1

1. The dependent variable is treatment result (Not cured including cases transferred to multidrug-resistant treatment, lost follow-up, and death due to other diseases).

2. The independent variable is the mutation status of virulence-related genes in Lineage2 of *Mycobacterium tuberculosis* (MTB) that is meaningful in univariate analysis.

**Table 4.** Multivariate analysis of the impact of Lineage 2 *Mycobacterium tuberculosis* virulence-related gene mutations on the prognosis of tuberculosis patients.

					95% C.I. for EXP(β)	
	β	Wals	P	Exp (β)	Lower	Upper
aftB	−0.6526	<0.0001	>0.9999	0.5207	<0.0001	–
eccC4	20.0430	<0.0001	0.9972	5.0651 × 10^8^	<0.0001	–
espE	2.6115	6.1483	0.0132	13.6200	1.7285	107.3201
fadE29	1.9638	5.2548	0.0219	7.1262	1.3294	38.1997
mbtB	−19.2791	<0.0001	0.9997	<0.0001	<0.0001	–
mas	19.5769	<0.0001	0.9995	3.1780 × 10^8^	<0.0001	–
mbtI	2.6969	4.2951	0.0382	14.8340	1.1577	190.0784
mmpL10	19.2722	<0.0001	0.9996	2.3433 × 10^8^	<0.0001	–
mprB	19.5261	<0.0001	0.9990	3.0204 × 10^8^	<0.0001	–
PPE26	0.2733	0.1776	0.6734	1.3143	0.3687	4.6850
ppsA	−0.6918	3.4529	0.0631	0.5007	0.2413	1.0386
Value of propensity scores	7.3188	7.3581	0.0067	1508.4137	7.6190	2.9864 × 10^5^
Constant	−41.2386	<0.0001	0.9960	<0.0001	–	–

1. Multivariate analysis used the forced entry method of multivariate logistic regression model. The independent variable is the virulence-related genes mutation status of Lineage2 *Mycobacterium tuberculosis*. The covariate is the propensity score value of resistance gene. the dependent variable is the treatment outcome of tuberculosis patients infected with Lineage2 *Mycobacterium tuberculosis*.

2. The model is statistically significant (χ2 = 51.1485, *p* < 0.0001). The lipF gene did not enter the model.

3. β, Wals, P, Exp (β), and 95% C.I. for EXP(β) represent coefficient, Statistical value, probability(*p* value), Odds Ratio (OR), and 95% confidence interval for Odds Ratio (95% CI of OR), respectively.

**Table 5.** Generalized multifactor dimensionality reduction analysis of the impact of Lineage 2 *Mycobacterium tuberculosis* virulence-related gene mutations on the prognosis of tuberculosis patients.

Model	Training Bal. Acc.	Testing Bal. Acc.	Sign Test(P)	CV Consistency
fadE29	0.5406	0.5033	5(0.6230)	7/10
espE and fadE29	0.5728	0.5542	6(0.3770)	9/10
mbtI and espE and fadE29	0.5921	0.5934	8(0.0547)	10/10

1. Generalized Multifactor Dimensionality Reduction (GMDR) was used to evaluate gene–gene interaction.

2. Model represents different combination patterns among significance virulence-related genes in multivariate logistic regression analysis of Lineage 2 *Mycobacterium tuberculosis*.

3. Training Bal. Acc, Testing Bal. Acc., Sign Test(P) and CV Consistency represent model training set, model test set, Statistical value (Probability (*p* value)), and cross-validation consistency, respectively.

**Table 6.** Prediction of the impact of virulence-related gene mutations on protein function.

Type	Gene	Variant	PROVEAN score	Prediction (cutoff = −2.5)
SNP	espE	L21V	−1.215	Neutral
SNP	espE	N87K	−1.43	Neutral
SNP	espE	R104W	0.115	Neutral
SNP	espE	P223R	−0.299	Neutral
SNP	espE	Q362R	−1.53	Neutral
SNP	fadE29	T139S	−3.783	Deleterious
SNP	fadE29	A202G	−2.243	Neutral
SNP	fadE29	I288V	0.412	Neutral
SNP	fadE29	R46Q	−2.61	Deleterious
SNP	mbtI	C50R	5.463	Neutral
SNP	mbtI	R98P	−1.344	Neutral
SNP	mbtI	D162G	−0.982	Neutral

Mutation function prediction using PROVEAN scoring method.

**Table 7.** Assignment table of multifactorial logistic regression analysis (L3).

Variable	Value	
Dependent variable	Cured = 0, Not cured = 1	
Independent variable		
eccE2	Wild type = 0, Mutation type = 1	
purC	Wild type = 0, Mutation type = 1	
PPE68	Wild type = 0, Mutation type = 1	

1. The dependent variable is treatment result (Not cured including cases transferred to multidrug-resistant treatment, lost follow-up, and death due to other diseases).

2. The independent variable is the mutation status of virulence-related genes in Lineage3 of *Mycobacterium tuberculosis* (MTB) that is meaningful in univariate analysis.

**Table 8.** Multifactorial logistic regression analysis of the effect of virulence gene mutations on treatment outcome in tuberculosis patients (L3).

					95% C.I. for EXP(β)	
	β	Wals	P	Exp (β)	Lower	Upper
PPE68	41.1622	<0.001	0.9989	7.5254 × 10^17^	<0.001	–
purC	−17.6424	<0.001	0.9991	<0.001	<0.001	–
eccE2	20.9383	<0.001	0.9989	1.2399 × 10^9^	<0.001	
Constant	−3.2958	10.4746	0.0012	0.0370	–	–

1. Multivariate analysis used the forced entry method of multivariate logistic regression model. The independent variable is the virulence-related genes mutation status of Lineage3 *Mycobacterium tuberculosis*, and the dependent variable is the treatment outcome of tuberculosis patients infected with Lineage3 *Mycobacterium tuberculosis*.

2. The model is statistically significant (χ2 = 13.9472, *p* = 0.0030).

3. β, Wals, P, Exp (β), and 95% C.I. for EXP (β) represent coefficient, Statistical value, probability (*p* value), Odds Ratio (OR), and 95% confidence interval for Odds Ratio (95% CI of OR), respectively.

## Discussion

The fight against tuberculosis remains a pressing global health concern, inflicting significant suffering on patients and exerting substantial economic strain on countries alike [43–46]. To achieve the ambitious goal of eliminating tuberculosis by 2035, as proposed by the WHO, it is crucial to prioritize the study of *M. tuberculosis*, including its transmissibility, pathogenicity, and drug resistance. Importantly, the dissemination, pathogenicity, and drug resistance of different strains of tuberculosis are closely linked to the variation and evolution of their virulence factors [47,48]. Therefore, investigating the differences in virulence factors and genes related to virulence in *M. tuberculosis* is of utmost importance in tuberculosis research. Recent advancements in molecular biology and bioinformatics have empowered the exploration of *M. tuberculosis* virulence factors’ variations and evolutionary trajectories at the genetic and molecular levels [49–51]. This progress has led to the identification of over 80 virulence factors influencing pathogenicity, drug resistance, and transmissibility, shedding light on the regulatory functions and evolutionary dynamics across different *M. tuberculosis* lineages [34,52–54]. However, numerous unanswered questions persist, demanding further investigation, such as identifying unknown virulence factors, understanding the mechanisms and disparities in the action of existing virulence factors across lineages, assessing the impact of virulence factor variation on diagnosis, treatment outcomes, and forecasting evolutionary trends. This study focused on a region where multiple *M. tuberculosis* lineages coexist, aiming to uncover variances in virulence factors and their related gene variations among these lineages and their significance for patient diagnosis, treatment, and prognosis. Consequently, our findings provide a valuable reference for future research elucidating *M. tuberculosis* virulence factor mechanisms and evolutionary trajectories.

In this study, we conducted WGS and bioinformatics analysis of 457 *M. tuberculosis* strains, revealing mutation in 71 virulence factors, displaying distinct mutation rates. Among these, 23 virulence factors exhibited notably high mutation rates, with nine virulence factors, namely ESAT-6 secretion system-1 (ESX-1), ESX-2 (T7SS), ESX-3, Mce1, Mce2, Mce3, NuoG, Phthiocerol dimycocerosate (PDIM), and PhoP, showing a 100% mutation rate. Based on their functional classification, ESX-1, ESX-2, and ESX-3 belong to the secretion system; Mce1, Mce2, and Mce3 to the Mammalian cell entry (mce) operons; NuoG to Anti-apoptosis factors; PDIM to Cell surface components; and PhoP to Regulation. These findings underscore prevalent mutations in M. tuberculosis virulence factors in Urumqi, further supporting the notion that gene exchange and gene mutation in *M. tuberculosis* are more active in areas harboring multiple lineages. Notably, the identification of these nine virulence factors with a 100% mutation rate has been associated with the pathogenicity, infectivity, or drug resistance of *M. tuberculosis*. Animal experiments have demonstrated that ESX-1 plays a role in promoting pathogen replication by remodeling the intracellular environment. Additionally, it can disrupt phagosomal membranes, activate various cytoplasmic immune sensing pathways, and stimulate cytokine secretion and autophagy [55]. Conversely, ESX-3 contributes to metal homeostasis, with mutants exhibiting siderophore-bound iron uptake defects, leading to a significant accumulation of cell-associated mycobacteria siderophore [56]. The ESX-5 system, particularly the EccB5 protein, is crucial for type VII secretion, which is vital for growth and virulence [57]. Moreover, the MCE complex, another important virulence factor, facilitates the transport of fatty acids and cholesterol across impermeable membranes [58]. The MCE operon encodes six different MCE proteins, which together with inner membrane permeases form a complex that spans the mycobacterial cell wall. This complex is primarily responsible for the attachment and entry of the pathogen into host cells [59]. The virulence factor NuoG has also been associated with the resistance of the new anti-tuberculosis drugs bedaquiline and clofazimine. Additionally, PDIM can inhibit the inflammatory response of macrophages to *M. tuberculosis* [60,61]. Furthermore, the inactivation of the virulence factor phoP significantly reduces the proliferation of *M. tuberculosis* in both in vitro and in vivo infection models, emphasizing its importance in the PhoPR two-component system [62]. These findings underscore the multifaceted potential trajectories for future *M. tuberculosis* virulence factor variation and evolution in Urumqi, necessitating ongoing surveillance and understanding of these trends.

Further analysis of the differences in the mutation status of virulence factors among different lineages of *M. tuberculosis* encompassed 457 strains, revealing three predominant lineages: Lineage2, Lineage3, and Lineage4, consistent with the findings of Chen H et al [63]. Further analysis unveiled disparities in the mutation rates of 27 virulence factors across these lineages. Notably, Lineage2 exhibited doubled mutation rates for LipF and Mce4 compared to Lineage3 and Lineage4. LipF and Mce4 implicated in regulating host macrophage pH and facilitating host cell adhesion and entry [58,59,64,65], align with Lineage2’s recognized heightened virulence and transmissibility due to virulence factor mutations. The high mutation rate of LipF and Mce4 in Lineage2 could also contribute to the increased toxicity and transmissibility [21–26]. Animal experiments have also supported the notion that LipF’s regulation of the pH value in the macrophage environment provides a more favorable external environment for the survival of M. tuberculosis [64,65]. Therefore, monitoring LipF and Mce4 variation could potentially hinder *M. tuberculosis* survival within macrophages, indirectly restricting its proliferation within hosts. Moreover, Lineage3 exhibited over threefold higher mutation rates in ABC transporter, Antigen 85, DevRS, lipid phosphatase, and PE/PE-PGRS compared to Lineage2 and Lineage4. Lineage3, predominantly found in India and Central Asia, and occasionally in Xinjiang, northwest China [63], may play a pivotal role in multi-drug-resistant tuberculosis spread, potentially breaching geographical barriers and posing global dissemination risks [66]. These virulence factors, namely ABC transporter, Antigen 85, and DevRS, have also been demonstrated to be associated with the replication and survival of *M. tuberculosis*, the formation of branch membranes and the survival of mycobacteria under hypoxic conditions [67–69], underscoring their crucial role in the survival and reproduction of *M. tuberculosis* in the host.

The specific function of PE/PE-PGRS is currently unclear and requires further study. Nonetheless, it is hypothesized that this factor could be associated with the immune escape of *M. tuberculosis* [70]. In this study, the mutation rates of these virulence factors were higher in Lineage3 compared to Lineage2 and Lineage4, which are widely distributed globally. Furthermore, if Lineage3, a potential driver of the multi-drug-resistant tuberculosis epidemic, does indeed spread globally, it could potentially give rise to novel drug-resistant *M. tuberculosis*. Conversely, Lineage4 showcased fewer mutations, primarily Nitrate/nitrite transporter, Tryptophan synthesis, and Tyrosine phosphatase compared to Lineage2 and Lineage3. Recent studies have associated the Nitrate/nitrite transporter, Tryptophan synthesis, and Tyrosine phosphatase with drug resistance in *M. tuberculosis*. These mutations, associated with heightened drug resistance potential, demand heightened surveillance against emerging drug-resistant strains [71–73]. This highlights the need for vigilance in monitoring the emergence of new drug-resistant strains of *M. tuberculosis* and emphasizes the importance of prevention and control measures for drug-resistant tuberculosis. Additionally, within Lineage 2, sublineage 2.2.1 manifested higher virulence factor mutation rates compared to Lineage 2.2.2, signaling the dominance of Ancestral Beijing sublineages in Urumqi and the possibility of their transition to modern Beijing sublineages. According to research conducted in southern China by Ajawatanawong P et al., the evolutionary transition from the ancestor to the modern Beijing sublineage may have occurred gradually in southern China, wherein multiple ethnic groups exist [74], allowing co-evolving sublineage cycles that culminated in the modern Beijing strain’s emergence [74]. Similarly, in Xinjiang, the coexistence of multiple lineages of *M. tuberculosis* and the frequent movement of people from various countries provides favorable conditions for the transformation of Ancestral Beijing sublineages into modern Beijing sublineages [74]. However, more evidence is needed to confirm this hypothesis. Modern Beijing sublineages are recognized for heightened virulence and transmissibility, posing new challenges if this hypothesis proves valid, necessitating proactive tuberculosis prevention and control measures in Urumqi and across Xinjiang. Although the differences in mutation rates among the remaining 17 virulence factors among each subtype are small, their significance should not be underestimated.

To investigate the variation status of each virulence factor-related gene and the variation differences between different lineages, we conducted an analysis on the 211 detected virulence factor-related genes with variation. Following the VFDB species classification standards, we observed that a majority (50.71%) of the mutated genes were categorized as defensive virulence factors, indicating potential adaptation of *M. tuberculosis* toward the human host. However, the PHI-based classification revealed that 89.10% of these genes remain classified as unknown, unveiling considerable gaps in our understanding of bacterium–host interactions. Our analysis identified a total of seven virulence factor-related genes (nuoG, eccE3, PE35, eccA2, eccA3, mycP4, and mce2A) that were consistently mutated across all samples. Notably, the NuoG gene mutation rate was 100.00% in Lineage2 and Lineage3. It has been previously suggested that the NuoG gene may be associated with resistance to new anti-tuberculosis drugs such as bedaquiline and clofazimine. Mutations in this gene could potentially contribute to the increased resistance of Lineage2 and Lineage3 to these drugs [60]. Additionally, the eccE3 gene and PE35 gene mutation rates were found to be 100.00% in Lineage3 and Lineage4. The eccE3 gene, involved in the secretion system, and the PE35 gene, encode a secretion system substrate protein, play crucial roles in host–pathogen interactions. The ATPase domain in the EccC3 coupling protein is particularly important for secretion, while PE35 not only encodes immunogenic proteins that enhance the pathogen response but also forms complexes that can enhance multiple responses. This serves as a compensatory mechanism for the loss of genome content due to reductive evolution [75,76]. The mutation rate of eccA2, eccA3, and mycP4 genes in Lineage3 was 100.00%. According to VFDB classification and Crosskey TD research, the Ecc series genes and mycP series genes are conserved sequences, vital to secretion in the ESX system [77]. Furthermore, van Winden VJC et al. reported that while the MycP series genes are associated with membrane complexes, they are not part of the complexes. Instead, they stabilize other membrane components or play a role in processing secreted substrates [78]. These findings suggest that the mutation of the secretion system in Lineage3 tuberculosis could shape its future evolutionary trend. Among the virulence factor-related genes of *M. tuberculosis* in Lineage4, we only detected a 100.00% mutation rate in the mce2A gene, which belongs to the MCE operon and is primarily involved in the adhesion and entry of *M. tuberculosis* into host cells [59]. Further analysis of the mutational differences among 211 virulence factor-related genes in different lineages revealed that the mutation rates of 86 genes varied among Lineage2, Lineage3, and Lineage4. Notably, Lineage2 and Lineage3 exhibited significantly higher numbers of mutated virulence factor-related genes compared to Lineage4. This suggests more active gene exchange between Lineage2 and Lineage3 prevalent in Urumqi, indicating a potential evolutionary trajectory toward increased virulence, transmissibility, and drug resistance. However, the continuation of this active gene exchange and its consequences remain uncertain.

To investigate the potential impact of virulence factors and virulence factor-related gene variants of *M. tuberculosis* in Urumqi on treatment outcomes, we conducted factor analysis. Our findings revealed that only the espE, fadE29 and mbtI genes significantly influenced the treatment outcome of patients infected with Lineage2 *M. tuberculosis*, without interaction. However, no mutations in virulence factor-related genes were found to affect patients infected with Lineage3 and Lineage4 *M. tuberculosis*. Notably, the espA-espC-espD operons, associated with ESX-1 secretion, play a crucial role in the interaction between pathogens and host immune cells, and the optimal functioning of ESX-1 requires the unligated espACD operon [79]. Therefore, mutations in espE could potentially enhance the secretion of EspB or its ability to bind PA and PS, thereby increasing the virulence of *M. tuberculosis* and influencing patient treatment outcomes [80]. The gene fadE29 plays a crucial role in the formation of heteromeric acyl-coenzyme A (acyl-CoA) dehydrogenase FadE28-FadE29. Studies have demonstrated that this heteromeric enzyme, produced by the intracellular growth (igr) operon of *M. tuberculosis*, is associated with bacterial pathogenicity and persistence in macrophages and mice [81]. Consequently, mutations in the fadE29 gene could hinder the effectiveness of tuberculosis treatment by allowing the infection to persist in patients. Similarly, another study revealed that the Mg2 ± dependent *M. tuberculosis* salicylate synthase (MbtI) is a vital enzyme involved in siderophore biosynthesis, displaying no equivalent enzyme in mammals [82]. Therefore, mutations in MbtI may result in resistance to MbtI inhibitor anti-tuberculosis drugs, which can impede the treatment of tuberculosis [83]. Although previous studies have identified potential reasons for the impact of espE, fadE29 and mbtI gene mutations on the treatment outcomes of patients infected with Lineage2 *M. tuberculosis*, further research is necessary to elucidate the presence of other contributing factors, if any. It is worth noting that drug resistance genes may have an impact on the results when exploring the relationship between virulence-related genes and treatment outcomes. Therefore, we should consider the control of drug resistance gene interference during analysis.

While our study delineates differences in virulence factor distribution and mutation rates among *M. tuberculosis* lineages and their implications for treatment outcomes, the lack of experimental animal evidence limits the robustness of our findings. We attempted to explain the observed phenomena based on previous research and have found some supporting evidence. Nevertheless, the strength of this evidence is not as high as that obtained through animal experiments. Therefore, our next step involves designing corresponding animal verification experiments to strengthen our conclusion based on the phenomena observed in this study.

## Conclusion

In this study, we unveiled variations in mutations of virulence factors and virulence factor-related gene mutations across different lineages of *M. tuberculosis*. Notably, patients infected with Lineage2 *M. tuberculosis* and exhibiting mutations in the espE, fadE29 and mbtI genes are prone to unfavorable treatment outcomes. These findings hold significant implications for understanding the evolution, transmissibility, drug resistance, and pathogenicity of *M. tuberculosis* virulence factors in regions with diverse lineages and high population mobility. Moreover, they offer crucial insights for global tuberculosis prevention and control efforts, new drug research and development, and the pursuit of the WHO’s 2035 goal of eradicating tuberculosis. To bolster our findings, we aim to conduct animal verification experiments based on the observations made in this study, seeking more definitive evidence.

## Supplementary Material

Clean copy of Supplementary 7 - QVIR-2024-0025.R1.xlsx

Clean copy of Supplementary 3 - QVIR-2024-0025.R1.xlsx

Clean copy of Supplementary 5 - QVIR-2024-0025.R1.xlsx

Clean copy of Supplementary 1- QVIR-2024-0025.R1.xlsx

Clean copy of Supplementary 6 - QVIR-2024-0025.R1.xlsx

Clean copy of Supplementary 4 - QVIR-2024-0025.R1.xlsx

Revised Supplementary2.xlsx

## Data Availability

Anonymous data and genetic projects and biological samples data that support the findings of this study are available the National Drug Resistance Surveillance Database (NDRS, https://www.carss.cn/) and National Microbiology Data Center (NMDC) (https://nmdc.cn/resource/genomics/project/detail/NMDC10018716 and https://nmdc.cn/resource/genomics/sample/detail/NMDC20278962, Project number: NMDC10018716 and NMDC20278962) and other relevant raw data are available in Science Data Bank (SDB) (DOI: 10.57760/sciencedb.28437).
